# SSR-Based Analysis of Genetic Diversity and Resistance to Barley Scald and Net Blotch in a Collection of Barley from Kazakhstan

**DOI:** 10.3390/genes17030261

**Published:** 2026-02-25

**Authors:** Yuliya Genievskaya, Akerke Maulenbay, Alibek Zatybekov, Saule Abugalieva, Yerlan Turuspekov

**Affiliations:** 1 Laboratory of Molecular Genetics, Institute of Plant Biology and Biotechnology, Almaty 050040, Kazakhstan; julia.genievskaya@gmail.com (Y.G.); alexbek89@mail.ru (A.Z.); absaule17@gmail.com (S.A.); 2 Laboratory of Phytosanitary Safety, Research Institute of Biological Safety Problems, National Holding “QazBioPharm”, Gvardeisky 080409, Kazakhstan; a.maulenbay@biosafety.kz

**Keywords:** *Hordeum vulgare* L., disease resistance breeding, *Rhynchosporium commune*, *Pyrenophora teres* f. *teres*, microsatellites

## Abstract

**Background/Objectives**: Barley (*Hordeum vulgare* L.) is a major cereal crop in Kazakhstan; however, its productivity is frequently constrained by foliar diseases, particularly barley scald (BS) and net blotch (NB). Understanding the genetic diversity of barley germplasm and identifying resistance-associated alleles are essential for improving disease resistance in breeding programs. The objective of this study was to assess the genetic diversity and population structure in a collection of two-rowed spring barley accessions and to identify SSR alleles associated with resistance to BS and NB. **Methods**: A total of 86 two-rowed spring barley accessions were genotyped using 14 SSR markers. Phenotypic evaluation for BS and NB resistance was conducted under natural infection conditions across two environments in southeastern and southern Kazakhstan. Genetic diversity and population structure were analyzed using Neighbor-Joining (NJ) clustering, Principal Coordinate Analysis (PCoA), and STRUCTURE. Marker–trait associations were evaluated using MLM method. **Results**: Phenotypic assessments revealed significant environment-dependent variation in disease severity for both BS and NB. Population structure analyses consistently identified distinct genetic clusters within the collection. Seven significant (*p* < 0.05) allele–trait associations were detected. The *Bmac209* 176 bp allele exhibited the strongest association with NB severity at KRIAPG. Among the identified markers, *Bmag206* 246 bp was uniquely associated with reduced NB infection, whereas *Bmag206* 252 bp, *Bmag613* 176 bp, and *HvLEU* 186 bp were linked with higher susceptibility to NB and BS. **Conclusions**: The identified resistance- and susceptibility-associated SSR alleles provide useful diagnostic markers for marker-assisted selection and support the potential of allele pyramiding for developing barley cultivars with combined resistance to BS and NB. This study establishes a genetic framework to enhance barley disease resistance in Central Asian breeding programs.

## 1. Introduction

Barley (*Hordeum vulgare* L.) ranks fourth among global cereal crops in production, with an estimated 155 million metric tons harvested annually, serving as a vital staple for food, feed, and industrial uses, particularly in malting and brewing [1]. As a cool-season crop adapted to temperate climates, barley thrives in diverse agro-ecological zones, but its productivity is frequently compromised by biotic stresses, including fungal foliar diseases. Among these, barley scald (BS, caused by *Rhynchosporium commune*, formerly *Rhynchosporium secalis*) [2] and net blotch (NB, caused by *Pyrenophora teres* f. *teres*) [3] stand out as economically significant pathogens, capable of inflicting substantial yield reductions and degrading grain quality worldwide. BS manifests as diamond-shaped lesions with grayish-white centers and brown margins on leaves, disrupting photosynthesis and reducing grain filling [2,4], while NB produces characteristic net-like patterns of necrotic streaks, further exacerbating canopy damage under humid conditions [5]. In severe epidemics, these diseases can diminish yields by 30–40%, with additional losses in malt quality due to mycotoxin contamination and reduced kernel weight [2,3,4,5]. The prevalence of these pathogens is amplified by climate variability, including prolonged wet periods that favor spore dispersal [2,5], underscoring the need for resilient cultivars in contemporary agriculture.

The global impact of BS and NB is particularly acute in major barley-producing regions, where they erode the economic viability of cultivation. Yield losses close to 100% due to BS have been reported in Morocco, Ethiopia, Tunisia, Peru, Colombia, Bolivia, Ecuador, and Turkey, where barley is constantly grown without crop rotation [6,7]. Yield losses of 10–45% were documented and economic yield is lowered due to inferior grain quality for goods like malting barley [8].

Similarly, NB can cause typical yield losses of 10–40%, with the potential for total loss if susceptible cultivars are planted under extreme environmental conditions [9]. In the Northern Caucasus, the Northwestern and Central Non-Chernozem regions, the Southern Urals, the Russian Far East, and Belarus, NB is recognized as the predominant disease affecting barley [10]. In northern Kazakhstan, NB is one of the most widely spread fungal diseases, with severity up to 40% [11,12]. Effective management relies on an integrated approach, but fungicide applications are costly and environmentally burdensome, often exceeding 20% of production expenses in high-disease areas. Consequently, breeding for durable resistance emerges as the cornerstone of sustainable control, leveraging genetic diversity to fortify crops against these persistent threats.

In Kazakhstan, barley holds high importance as the second-leading cereal after wheat, supporting food security, livestock feed, and export revenues with a harvest approximating 2.6 million tons in 2023 [13]. The country’s vast steppes and semi-arid to continental climate in the northern and eastern regions—the prime barley belts—yielded an average of 1.68 tonnes per hectare in 2024 [14], but production is frequently curtailed by abiotic extremes and biotic stresses. The most intensive manifestation of NB has been reported in the dry-steppe and foothill-steppe zones, where environmental conditions favor recurrent pathogen development [15]. In northern Kazakhstan and adjacent regions of Russia, the disease develops annually, with its spread reaching 80–90% and infection severity exceeding 40% in certain years [12]. Previous studies conducted in southeastern Kazakhstan involving 41 domestic spring barley genotypes revealed moderately resistant reactions to NB (5–20% severity) and to BS (10–20% severity) [16]. In northern Kazakhstan, local spring barley genotypes demonstrated up to 70% severity of NB at the grain filling stage of growth [17]. All of that suggests high possible risks for the decrease in barley grain yield and quality in the case of epiphytotic development of these diseases. Thus, fortifying local germplasm against BS and NB is imperative for sustaining yields, enhancing export competitiveness, and bolstering resilience in this agronomically critical sector.

The quest for resistance has historically relied on phenotypic selection, yet this approach is labor-intensive and confounded by environmental interactions, prompting a shift toward molecular breeding paradigms. Molecular markers revolutionize breeding by enabling early, precise genotyping, independent of field expression. In barley, markers facilitate quantitative trait locus (QTL) mapping [18,19,20], genome-wide association studies (GWAS) [21,22], and marker-assisted selection (MAS) [23,24] for disease resistance, accelerating the introgression of resistance to BS and NB into breeding lines. Recent studies in Kazakhstan have applied GWAS and QTL mapping to dissect adult plant resistance to stem rust [25] and powdery mildew [26], providing insight into the genetic architecture of disease resistance in local barley germplasm. For fungal diseases, where resistance is often polygenic and quantitative [27,28], markers bridge the genotype-phenotype gap, permitting pyramiding of favorable alleles for durable protection.

Among all DNA markers, simple sequence repeat (SSR) markers have proven instrumental in dissecting the genetic architecture of barley resistance to BS and NB, enabling precise QTL mapping and marker-assisted selection for durable disease management. In BS studies, SSRs facilitated the identification of three major QTLs introgressed from wild barley (*Hordeum v.* ssp. *spontaneum*), explaining up to 16.5% of phenotypic variance in field trials across diverse environments [29]. Similarly, fine mapping of the *Rrs2* resistance gene on chromosome 7HS utilized SSR-flanking markers to narrow the locus to 0.08 cM, supporting diagnostic tools for pyramiding resistance alleles [30]. For NB, SSR-based linkage maps in doubled-haploid populations pinpointed key QTLs like *QRpt6* on chromosome 6H, accounting for 89% of seedling resistance variation, while additional loci on 4H and 7H were tagged for both net and spot forms, enhancing breeding efficiency in susceptible germplasm [31]. These applications underscore SSRs’ codominant polymorphism and genome-wide coverage, accelerating the introgression of polygenic resistance to mitigate yield losses.

Despite these advances, gaps persist in applying SSRs to Central Asian collections, where scald and net blotch co-occur amid abiotic stressors like drought, necessitating integrated phenotyping-genotyping strategies. Prior studies highlight SSR-linked associations in global panels, but local accessions from Kazakhstan—shaped by unique evolutionary pressures—demand empirical validation for practical deployment.

To date, comprehensive studies integrating SSR-based genotyping with multi-environment field resistance data to identify diagnostic alleles for BS and NB resistance in local barley germplasm remain limited. To address these challenges, the present study evaluates a diverse collection of barley genotypes from Kazakhstan using SSR markers. Specific objectives are: (1) to quantify genetic diversity and delineate population structure, elucidating evolutionary relationships and adaptive potential; (2) to assess resistance of local barley cultivars and lines to BS and NB; and (3) to identify marker-trait associations linking SSR alleles to field-evaluated resistance phenotypes for scald and net blotch. By bridging molecular insights with phenotypic resilience, this work aims to empower Kazakhstan’s breeders in developing robust, disease-tolerant cultivars, securing sustainable production amid escalating biotic threats.

## 2. Materials and Methods

### 2.1. Barley Germplasm and Resistance Assessment in the Field

The germplasm used in this study was collected from six breeding organizations in Kazakhstan and consisted of 86 two-rowed spring barley cultivars and breeding lines (Supplementary Table S1). The barley collection was evaluated for resistance to two major foliar fungal diseases, BS and NB.

Field experiments were conducted at two locations. In 2022, trials were established at the Kazakh Research Institute of Agriculture and Plant Growing (KRIAPG), southeastern Kazakhstan (43.229402° N, 76.699168° E). In 2024, the collection was evaluated at the Research Institute of Biological Safety Problems (RIBSP), southern Kazakhstan (43.576476° N, 75.213618° E). At each location, the experiments were arranged in a randomized complete block design with two replications. Meteorological data of the vegetation period, including monthly temperature, precipitation, relative humidity, and sunshine hours at KRIAPG and RIBSP, are provided in Supplementary Table S2.

Each accession was grown in a single 1 m long row with a row spacing of 15 cm. No artificial inoculation was applied, and disease development occurred under natural field infection conditions. Disease severity was assessed at the adult plant stage, from flag leaf emergence to heading, when symptoms of both diseases were fully expressed. Visual assessments were conducted repeatedly during this period, and for each accession, the highest disease severity score recorded across the two replications was used for further analyses. Disease severity was visually estimated using a 1–100% scale, based on the proportion of necrotic and chlorotic leaf area, following standard methodologies for cereal foliar diseases [32,33].

### 2.2. SSR Genotyping

A total of 14 SSR markers were used for genotyping, including seven markers associated with resistance to BS and seven markers associated with resistance to NB (Table 1).

Genomic DNA was isolated from 5-day-old barley seedlings using the DNeasy Plant Pro Kit (QIAGEN, Hilden, Germany), with three biological replicates per accession. PCR conditions were optimized to ensure high amplification efficiency and reproducibility. Amplifications were performed in a total reaction volume of 20 µL containing 20 ng of genomic DNA, 1 U of Taq DNA polymerase, 0.2 mM of each deoxyribonucleotide triphosphate (dNTP), 10 pM of each primer, 1.5 mM magnesium chloride (MgCl_2_), and 1× Taq reaction buffer. PCR amplification was carried out in a SimpliAmp Thermal Cycler (Thermo Fisher Scientific, Singapore, Singapore) with an initial denaturation at 94 °C for 3 min, followed by 40 cycles of denaturation at 94 °C for 30 s, annealing at the primer-specific temperature (Ta) for 50 s, and extension at 72 °C for 1 min 40 s, and a final extension at 72 °C for 5 min. Amplified products were separated by capillary electrophoresis using a QIAxcel Connect System (QIAGEN, Hilden, Germany) equipped with a QIAxcel DNA High Resolution Kit, QX Alignment Marker 15 bp/3 kb, and QX Size Marker (50 bp/1 kb). Sample analysis was conducted using the standard OH500 method with an injection time of 20 s.

### 2.3. Statistics

Statistical analyses and data visualization were performed using R version 4.5.0 [44]. Relationships between disease resistance parameters were evaluated using Pearson’s correlation coefficients. Correlation analyses and graphical visualization of correlation matrices were conducted using the R packages PerformanceAnalytics and ggplot2.

The genetic diversity of SSR loci and barley accessions were assessed using GenAlEx version 6.503 [45]. The following diversity indices were calculated: number of effective alleles (Ne), Shannon’s information index (I), polymorphism information content (PIC), and the percentage of polymorphic loci per accession (%P). Analysis of molecular variance (AMOVA) was performed to partition genetic variation within and among predefined groups and to estimate the relative contribution of different hierarchical levels to the total genetic diversity.

Population structure and genetic relationships among accessions were investigated using complementary analytical approaches. Principal coordinates analysis (PCoA) was conducted in GenAlEx based on genetic distance matrices. A neighbor-joining (NJ) tree was constructed using PAST4 software version 4 [46]. Bayesian clustering analysis was performed using STRUCTURE version 2.3.4 [47], with the number of assumed populations (K) ranging from 1 to 10, a burn-in period of 100,000 iterations, and 100,000 Markov chain Monte Carlo (MCMC) replications. The optimal number of genetic clusters was determined using CLUMPAK version 1 [48], which was applied to summarize and visualize STRUCTURE outputs and to identify the most likely K based on cluster stability and Prob(K) values. To integrate and compare clustering results obtained from different analytical methods, an alluvial diagram was constructed using the R package ggalluvial.

Associations between SSR alleles and resistance to BS and NB were evaluated using a mixed linear model (MLM) in GAPIT v3 [49], controlling for population structure (Q matrix) and kinship (K matrix). Alleles with low-frequency (<0.05) were removed from the analysis. Disease severity scores were compared among alleles for each SSR locus to identify those significantly associated with variation in resistance levels. Boxplots illustrating the distribution of disease severity scores across allele groups were generated using R.

## 3. Results

### 3.1. Field Resistance to BS and NB

**Figure 1:**
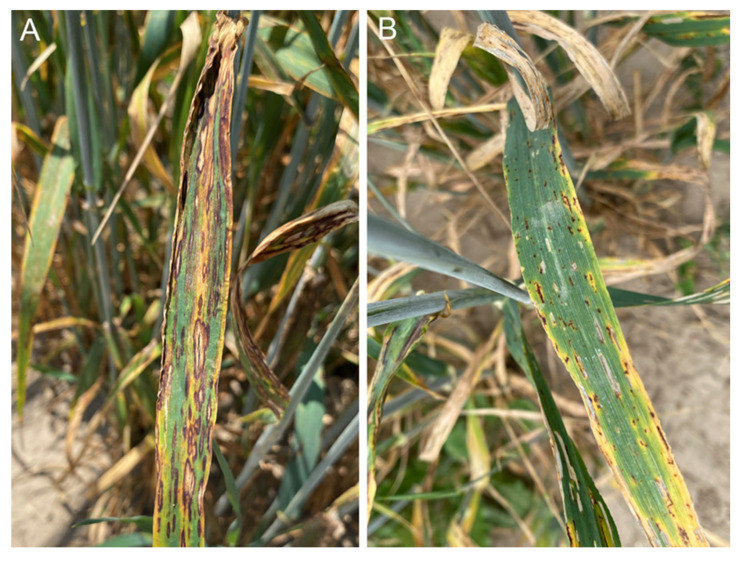
Visual assessment of plant reaction to barley scald in accession QB_190 at RIBSP (**A**) and net blotch in accession QB_154 at KRIAPG (**B**).

Phenotypic evaluation revealed clear differences in disease severity and variability between the two testing environments (Figure 1, Table 2).

For BS, disease severity at KRIAPG in 2022 ranged from 0 to 40%, with a mean of 17.8 ± 9.8%. Based on reaction types, 30 accessions were classified as immune (0%), 35 as resistant (1–10% affected leaf area), 10 as moderately resistant (11–25%), and 11 as moderately susceptible (26–50%). At RIBSP in 2024, BS severity showed a slightly wider range (0–50%) but a lower mean value of 14.8 ± 12.0%. Under these conditions, 66 accessions were immune, 6 were moderately resistant, and 12 were moderately susceptible. No susceptible reactions to BS were observed in either environment.

A similar environment-dependent pattern was observed for NB (Table 2). At KRIAPG, NB severity ranged from 0 to 30%, with a mean of 12.2 ± 5.1% and moderate variability (CV = 41.8%). Most accessions were immune (61 accessions), followed by resistant (16), moderately resistant (5), and moderately susceptible (4). In contrast, disease pressure at RIBSP was substantially higher, with severity values ranging from 0 to 60% and a mean of 29.0 ± 14.6%. Reaction types at RIBSP were more evenly distributed, including 10 immune, 31 resistant, 24 moderately resistant, 18 moderately susceptible, and 3 susceptible accessions.

Mean disease severity values across the two environments were 16.3 ± 10.9% for BS and 20.6 ± 9.9% for NB. Overall, BS severity was higher at KRIAPG, whereas NB development was favored under the environmental conditions at RIBSP, indicating contrasting responses of the two diseases to environmental variation.

**Figure 2:**
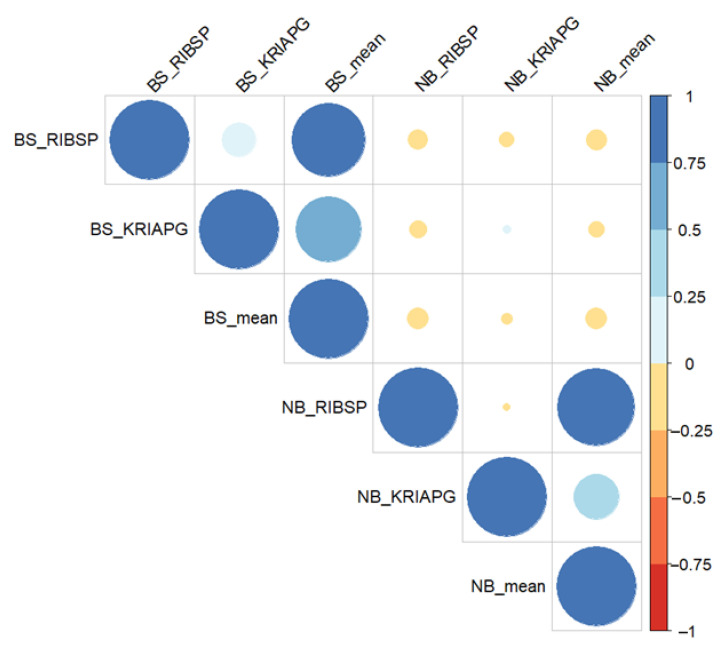
Correlation analysis between barley scald (BS) and net blotch (NB) severity score across two regions, including mean values. The size of each circle corresponds to *p*-value, while the color indicates the sign and magnitude of the correlation coefficient *r*. The color scale on the right defines the correlation strength, ranging from −1 (negative correlation) to +1 (positive correlation).

Correlation analysis revealed strong and significant associations between mean disease severity values and their corresponding environment-specific assessments for both diseases (Figure 2).

For BS, mean severity was strongly correlated with disease scores recorded at KRIAPG (*p* < 0.001) and RIBSP (*p* < 0.001). However, the direct correlation between BS severity values at KRIAPG and RIBSP was weak and not significant. A similar pattern was observed for NB: mean NB severity showed strong correlations with environment-specific scores at KRIAPG (*p* < 0.01) and RIBSP (*p* < 0.001), whereas no significant correlation was detected between NB severity values recorded at the two locations.

No significant correlations were observed between BS- and NB-related traits, either within individual environments or when mean values were compared. Collectively, these results demonstrate pronounced environment-dependent expression of resistance to BS and NB, with consistent relationships between environment-specific and mean disease severity values but limited agreement between environments. The observed phenotypic variation and clear differentiation among accessions provide a robust basis for subsequent analyses of SSR marker–trait associations.

### 3.2. Descriptive Statistics of SSRs

**Figure 3:**
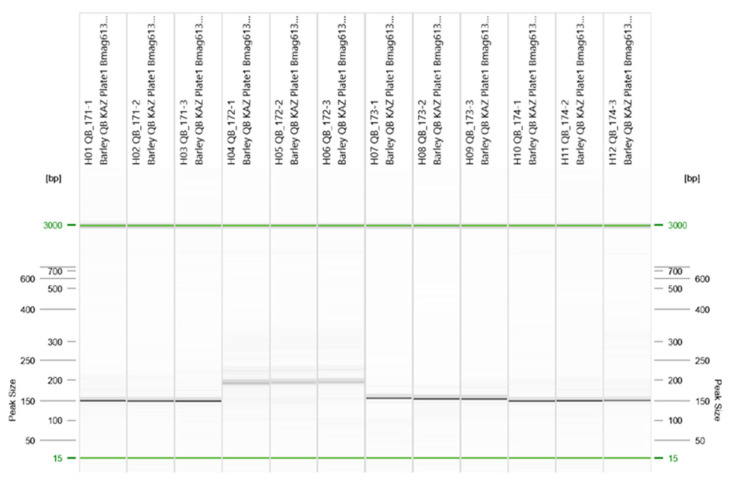
Digital gel electrophoresis profile of *Bmag613* locus in barley accessions (fragment). Alleles with sizes of 150, 194, 154, and 150 bp are shown from left to right; each allele size is represented by three independent replicates per barley accession. Green lines indicate alignment marker with 3000 bp and 15 bp sizes.

In total, 14 SSR markers associated with resistance to BS and NB were used to genotype the barley collection from Kazakhstan. Of these, 13 markers were polymorphic, whereas one marker (*Bmag381*) was monomorphic (Supplementary Table S3). A representative electropherogram profile is shown in Figure 3.

The genetic diversity of the 13 polymorphic SSR markers was assessed using four parameters, which are summarized in Table 3.

The Na ranged from 2 (*GBM1094*) to 12 (*Bmag206*), with a mean value of 6.69 ± 0.91. The Ne varied from 1.27 (*Bmag222*) to 6.93 (*Bmag206*), with an average of 3.50 ± 0.51, indicating pronounced differences in allele frequency distributions among loci. I-values ranged from 0.51 (*Bmag222*) to 2.12 (*Bmag206*), with a mean value of 1.32 ± 0.15, reflecting moderate to high levels of genetic diversity. PIC values ranged from 0.215 (*Bmag222*) to 0.854 (*Bmag206*), with an overall mean of 0.637 ± 0.021.

Highly informative loci (PIC ≥ 0.80) included *HVM03* (0.819), *Bmag606* (0.810), *Bmag206* (0.854), *Bmac209* (0.839), and *Bmag613* (0.802), whereas *Bmag222* and *GBM1094* exhibited low levels of polymorphism. Overall, the SSR marker set provided sufficient resolution for a reliable assessment of genetic diversity and supported subsequent analyses of population structure and marker–trait associations.

### 3.3. Genetic Diversity of Barley Accessions

The genetic diversity of the 86 barley accessions was assessed using the same set of 13 polymorphic SSR markers, and the results are summarized in Table 4. Overall, low levels of intra-accession polymorphism were observed across the barley collection.

The %P per accession ranged from 0.00 to 46.15%, with the highest value recorded for accession QB_145 (46.15%), followed by QB_189 (38.46%) and QB_147 and QB_165 (30.77%). Nineteen accessions showed no detectable polymorphism, while 28 accessions exhibited low levels of intra-accession polymorphism (%P < 10%). The Ne varied from 1.00 ± 0.00 in monomorphic accessions to 1.37 ± 0.12 in QB_145, with most accessions displaying Ne values between 1.06 and 1.12. Similarly, I ranged from 0.00 ± 0.00 to 0.29 ± 0.09, indicating generally low but variable genetic diversity within accessions.

**Figure 4:**
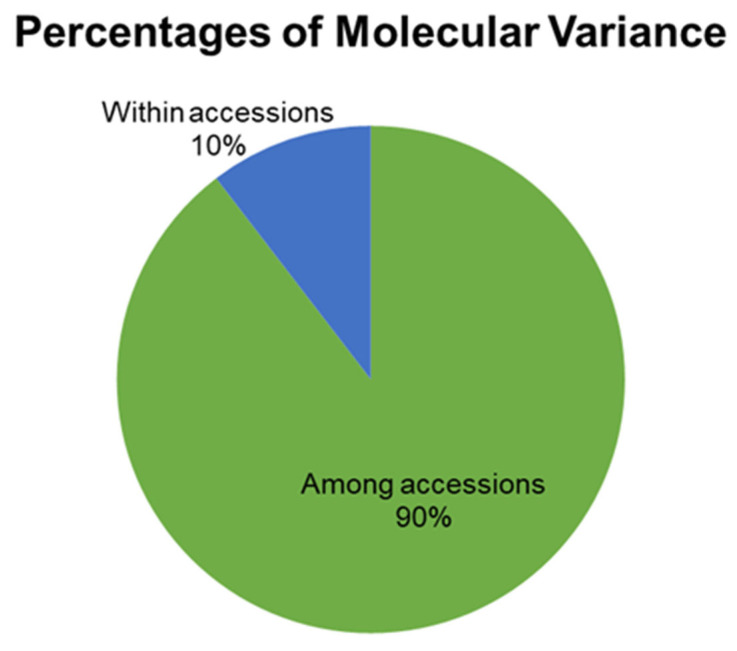
Percentages of molecular variance among and within the studied barley accessions (*p* < 0.001).

Consistent with these observations, AMOVA showed that the majority of genetic variation was partitioned among accessions (Figure 4).

Specifically, 90% of the total molecular variance was attributed to differences among accessions, whereas only 10% was explained by variation within accessions, with both components being highly significant (*p* < 0.001). Overall, these results indicate a predominantly homogeneous genetic structure at the accession level, while identifying a limited number of accessions with elevated intra-accession polymorphism.

### 3.4. Population Structure

**Figure 5:**
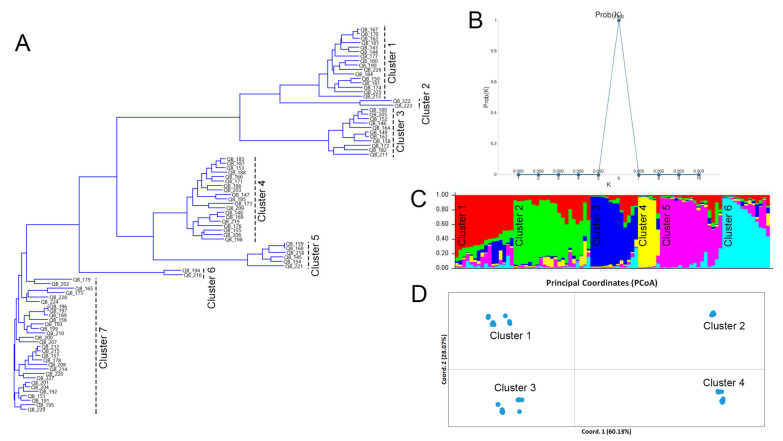
Population structure and genetic relationships of barley accessions based on SSR markers: NJ tree (**A**), Prob(K) values (**B**), STRUCTURE barplot at K = 6 (**C**), and PCoA (**D**).

The genetic architecture and population structure of the barley accessions were characterized using a complementary approach based on NJ tree analysis, Bayesian admixture clustering (STRUCTURE), and PCoA (Figure 5).

The NJ phylogenetic tree (Figure 5A) partitioned the accessions into seven distinct clusters, indicating pronounced genetic differentiation within the collection. The most likely number of genetic subpopulations was determined based on Prob(K) values, which showed a clear maximum at K = 6 (Figure 5B). Accordingly, the STRUCTURE barplot was generated at K = 6 (Figure 5C), illustrating the assignment of each accession to one of six inferred ancestral groups. The results of Bayesian clustering were supported by PCoA (Figure 5D), which provided a spatial representation of genetic relationships based on genetic distance matrices. The first two principal coordinates explained 88.20% of the total genetic variation, with Coordinate 1 accounting for 60.13% and Coordinate 2 for an additional 28.07%.

**Figure 6:**
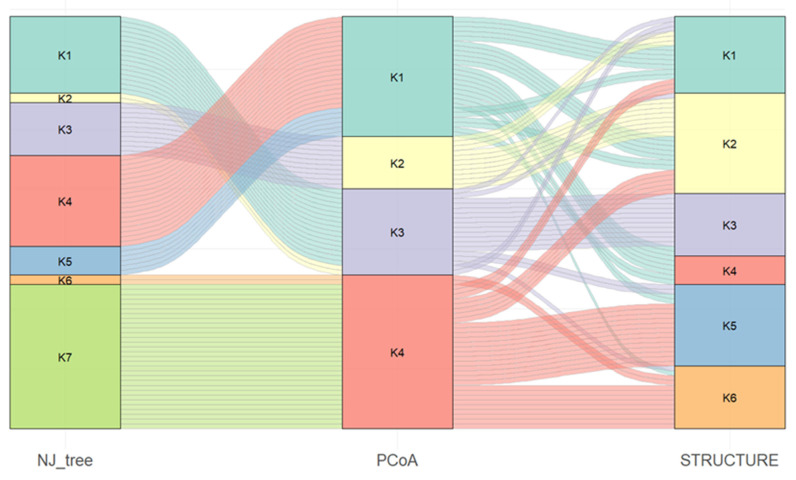
Alluvial diagram of cluster assignments across NJ tree, PCoA, and STRUCTURE analyses. The different colors correspond to different K values and represent individual genetic clusters inferred for each method.

To assess concordance among clustering methods, an alluvial diagram was constructed to track cluster assignments across the NJ tree, PCoA, and STRUCTURE analyses (Figure 6).

Clear correspondence was observed between several NJ tree and PCoA clusters. Specifically, NJ tree cluster K3 fully corresponded to PCoA cluster K2. In contrast, the remaining NJ clusters were consolidated into broader PCoA groupings: NJ clusters K1 and K2 were grouped within PCoA cluster K3, NJ clusters K4 and K5 within PCoA cluster K1, and NJ clusters K6 and K7 within PCoA cluster K4. The comparison between PCoA and STRUCTURE results revealed pronounced fragmentation of the largest PCoA clusters (K4 and K1), whose accessions were distributed across all six STRUCTURE-defined subpopulations.

Overall, all three analytical approaches captured similar broad patterns of genetic structure; however, the STRUCTURE analysis resolved a finer level of subdivision that was not fully apparent in the distance-based NJ tree and PCoA analyses.

Resistance levels to BS and NB were subsequently compared among clusters defined by the three analytical methods (Supplementary Figure S1). Substantial variation in mean NB severity was observed among clusters across all approaches. In the NJ tree grouping, clusters K6 and K7 exhibited the highest mean NB severity. Consistently, PCoA cluster K4 showed the highest mean NB severity among the four PCoA-defined groups. STRUCTURE analysis further differentiated resistance levels, with cluster K6 displaying the highest mean NB severity. In contrast, no significant differences in resistance to BS were detected among clusters identified by any of the three methods.

### 3.5. Associations Between Alleles and Resistance to BS and NB

Associations between SSR marker alleles and resistance to BS and NB, evaluated across two environments and as mean values, were assessed using MLM analysis. This analysis identified seven significant allele–trait associations (*p* < 0.05) for resistance to two diseases across environments (Table 5).

The strongest association was observed for *Bmac209_176* bp with NB resistance at KRIAPG. The allele *Bmag206_246 bp* showed negative effects on susceptibility to NB under mean and RIBSP conditions, indicating a protective effect by reducing infection. *Bmag206_252* bp was associated with BS resistance at RIBSP. *Bmag613_176* bp was linked to NB_mean, while *HvLEU_186* bp exhibited consistent positive effects on susceptibility BS across mean and KRIAPG environments.

**Figure 7:**
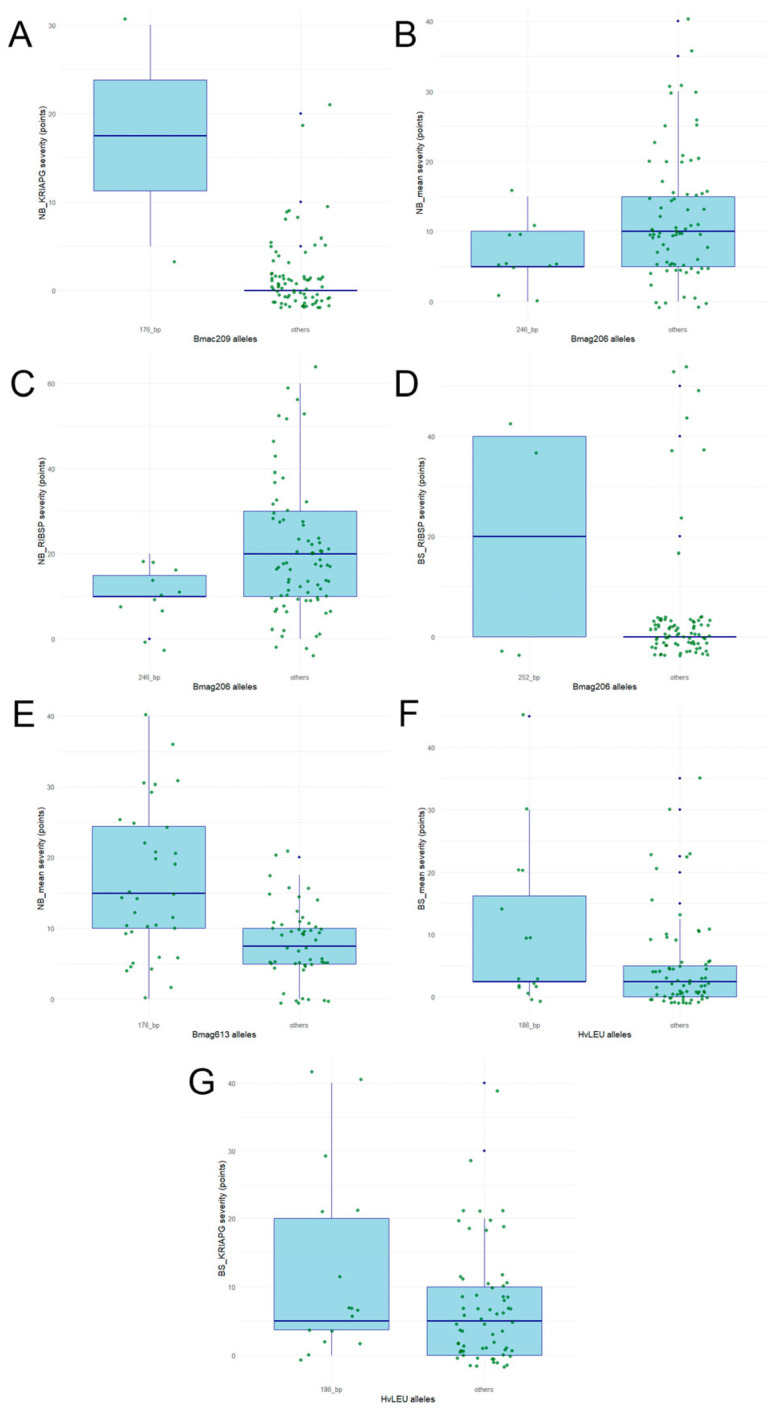
Severity of BS and NB in barley accessions carrying different alleles of significantly associated SSR markers (*p* < 0.05): *Bmac209* with NB_KRIAPG (**A**), *Bmag206* with NB_mean (**B**), *Bmag206* with NB_RIBSP (**C**), *Bmag206* with BS_RIBSP (**D**), *Bmag613* with NB_mean (**E**), *HvLEU* with BS_mean (**F**), and *HvLEU* with BS_ KRIAPG (**G**). Individual data points are plotted as jittered points, with green indicating the primary distribution of genotypes and blue highlighting statistical outliers exceeding 1.5 times the interquartile range.

The allelic variation at four SSR loci—*Bmac209*, *Bmag206*, *Bmag613*, and *HvLEU*—demonstrated a significant impact on the resistance levels of barley accessions against NB and BS across different phenotyping environments (Figure 7).

The *Bmac209* locus showed a robust association with NB severity at the KRIAPG site. Accessions carrying the 176 bp allele exhibited a markedly higher disease severity compared to those with alternative alleles, where the median severity remained near zero (Figure 7A). Similarly, the 176 bp allele at the *Bmag613* locus was associated with increased susceptibility to NB, with mean severity scores significantly elevated compared to other alleles (Figure 7E). The *Bmag206* marker was associated with disease response across multiple metrics, though its effect varied by allele. For NB_mean and NB_RIBSP, the 246 bp allele was associated with lower or comparable severity relative to the wider population (Figure 7B,C). In contrast, the 252 bp allele at this same locus was linked to a substantial increase in BS severity at the RIBSP site, whereas accessions lacking this allele were largely resistant (Figure 7D). The *HvLEU* locus primarily influenced BS response. Accessions harboring the 186 bp allele showed a wider range of infection responses and generally higher median severity scores for both BS_mean and BS_KRIAPG compared to accessions carrying alternative alleles (Figure 7F,G). This suggests that the 186 bp allele may serve as a marker for increased susceptibility to BS in the evaluated germplasm.

**Figure 8:**
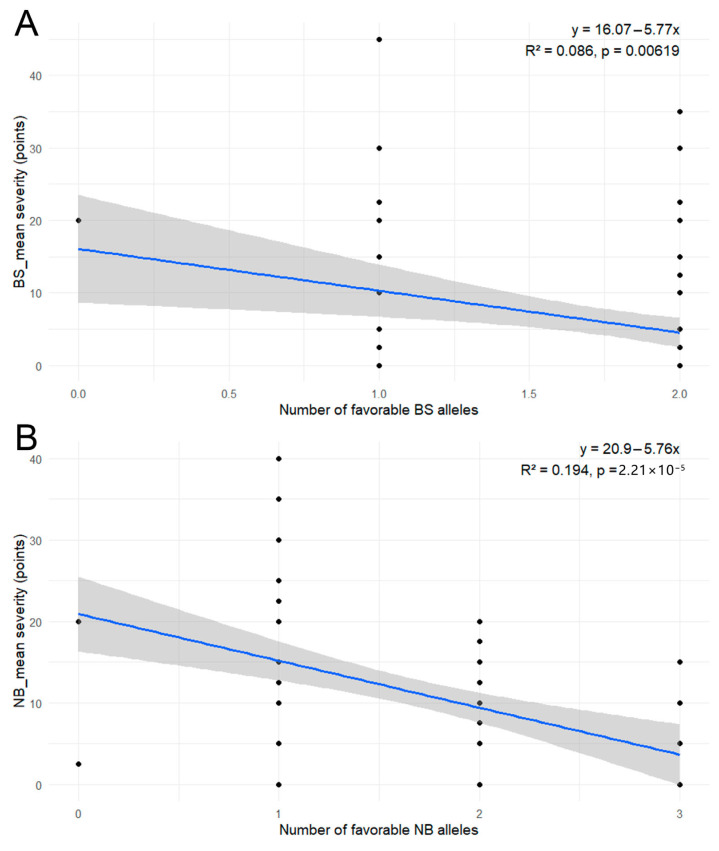
Regression analysis of NB resistance (**A**) and BS resistance (**B**) on favorable allele count.

Linear regression analysis was performed to evaluate the relationship between the accumulation of favorable alleles and the mean severity of BS and NB. For both diseases, a significant negative correlation was observed, indicating that an increase in favorable allele counts corresponds to a reduction in disease symptoms (Figure 8).

In the case of BS resistance (Figure 8A), the regression model was significant (*p* = 0.00619), although the R^2^ value of 0.086 suggests that allele count explains a relatively small portion of the total phenotypic variance. Conversely, the NB resistance (Figure 8B) demonstrated a stronger association (R^2^ = 0.194, *p* = 2.21 × 10^−5^), with the severity decreasing by approximately 5.5 points for every additional favorable allele. These data suggest that the identified in MLM analysis alleles contribute additively to enhancing host resistance.

## 4. Discussion

### 4.1. Environmental Effects and Phenotypic Expression of Resistance

Field evaluations revealed pronounced environment-dependent expression of resistance to both BS and NB. Differences in disease severity and reaction type distributions between KRIAPG and RIBSP indicated a strong influence of local environmental conditions on disease development, particularly for NB (Table 2). Higher NB severity at RIBSP (29.0% vs. 12.2%) is consistent with previous studies reporting that optimal temperature and higher humidity strongly affect net blotch epidemics [50,51]. In contrast, BS severity was slightly more pronounced at KRIAPG, reflecting pathogen-specific responses to environmental factors, as previously observed in multi-location barley trials [52], although the difference was small (17.8% at KRIAPG and 14.8% at RIBSP).

It should be noted that resistance was evaluated under natural infection without artificial inoculation. While this approach reflects realistic field conditions, pathogen pressure may vary spatially within and between locations, potentially contributing to phenotypic variability. In addition, the race composition of *R. commune* and *P. teres* populations was not characterized, and differences in virulence spectra between sites may have influenced resistance expression. Such environmental and pathogen heterogeneity may partly explain the absence of significant correlations between locations (Figure 2) and the environment-specific patterns observed. At the same time, strong correlations between environment-specific scores and mean values demonstrate that averaging across environments provides a reliable summary of overall disease response for quantitative resistance traits. These results highlight the importance of multi-environment testing for robust evaluation of disease resistance in barley breeding programs [53].

### 4.2. Genetic Diversity and Population Structure of Barley Germplasm from Kazakhstan

SSR-based analysis revealed moderate to high levels of genetic diversity at the marker level, with 5 highly informative loci (PIC ≥ 0.80) contributing to the resolution of genetic relationships among accessions (Table 3). Comparable levels of SSR polymorphism have been reported in barley collections of diverse geographic origin [54,55,56]. In contrast, genetic diversity within individual accessions was generally low (Table 4), reflecting the predominantly self-pollinating nature of barley and the breeding history of cultivated germplasm. AMOVA results confirmed that most molecular variation was distributed among accessions rather than within them (Figure 4), a pattern widely reported for barley breeding materials analyzed using SSR and SNP markers [57,58]. The limited intra-accession polymorphism observed (10%) supports the suitability of this collection for association analysis, as reduced within-line heterogeneity minimizes confounding effects on marker–trait relationships.

Although barley is predominantly self-pollinated, a small level of within-accession polymorphism was detected. This variation is primarily explained by residual heterozygosity and the heterogeneous nature of certain breeding materials that were not fully genetically stabilized at the time of sampling. Heterozygous SSR profiles were scored and retained following standard allele-calling procedures. Due to their low frequency, these genotypes had minimal impact on overall diversity estimates and population structure analysis.

Population structure analyses based on NJ tree clustering, PCoA, and STRUCTURE revealed clear genetic stratification within the collection (Figure 5). While the first two methods captured similar broad patterns, STRUCTURE resolved other subdivision, likely due to its ability to detect admixture and shared ancestry components. Partial concordance among clustering approaches and reassignment of accessions across methods have been commonly reported in barley and other crops [59,60]. Overall, the observed genetic diversity and clear population structure indicate a well-differentiated barley germplasm in Kazakhstan.

While the 14 SSR markers (13 polymorphic) utilized here represent a moderate panel, their selection was based on high polymorphism, reproducibility, and documented linkage to barley resistance loci. The multi-allelic nature of these markers provided substantial discriminatory power, evidenced by five loci with PIC ≥ 0.80 (Table 3). However, certain limitations regarding marker density must be acknowledged. Incomplete genome coverage and non-uniform distribution across the seven chromosomes may have precluded the detection of minor-effect loci or fine-scale population substructure [61,62]. Furthermore, the limited mapping resolution suggests that identified associations likely represent broad chromosomal segments rather than precise causal genes. Consequently, these marker–trait associations are considered preliminary signals. Future validation and fine-mapping should employ high-density SNP arrays to achieve the marker saturation required for higher genomic resolution.

### 4.3. SSR Marker–Trait Associations for BS and NB Resistance

The identification of seven significant allele–trait associations via MLM analysis (Table 5) underscores the complex genetic control of foliar disease resistance in the studied barley germplasm. Allele-level analysis revealed phenotypic contrasts associated with specific SSR alleles, suggesting linkage to functional resistance genes or closely linked QTLs, as commonly reported for SSR-based studies in barley [34,35,36,37,38,39,40,41,42]. Notably, the *Bmag206* 246 bp allele demonstrated a consistent protective effect against NB across both mean and site-specific environments, suggesting it may be linked to a stable, broad-spectrum resistance locus. Such stability is critical for breeding programs aiming for environmental resilience. In contrast, the *Bmac209* 176 bp marker exhibited the strongest association with NB at the KRIAPG site, which may indicate the presence of environment-specific QTLs or a response to localized pathogen races. Regression analysis confirmed additive effects of favorable alleles for both NB (R^2^ = 0.194) and BS (R^2^ = 0.086) (Figure 8).

Four SSR markers significantly associated with resistance to NB and BS in the current study were mapped within regions previously identified as QTLs (Table 1). The detection of significant associations in barley accessions from Kazakhstan extends the relevance of these markers to a geographically distinct breeding pool. Differences in trait specificity or strength of association observed for some markers may reflect variation in genetic background, pathogen populations, or environmental conditions, all of which are known to influence the expression of quantitative resistance [63]. These results highlight the importance of validating resistance markers under local field conditions prior to their application in breeding programs.

The significant negative correlation between the number of favorable alleles and disease severity suggests that resistance to both BS and NB is affected by an additive genetic architecture. This observed trend, where phenotypic severity decreases linearly with the accumulation of resistance loci, is a hallmark of quantitative resistance in barley and other crops [64]. The R^2^ values, particularly for NB resistance, indicate that while these specific loci are influential, a substantial portion of the phenotypic variance remains unexplained, likely due to the presence of minor-effect QTLs. Similar additive effects have been documented in studies of *R. commune*, where the pyramiding of multiple resistance genes provided significantly enhanced protection compared to single-gene lines [65]. Furthermore, the linear reduction in disease points observed here aligns with findings by Vatter et al. (2017) [66], who demonstrated that the stacking of favorable alleles for *P. teres* provides more durable field resistance than vertical resistance alone. These results reinforce the potential for MAS to systematically increase the frequency of these favorable alleles in barley breeding populations to combat multiple foliar pathogens simultaneously.

Future research may focus on integrating SNP-based analyses, fine-mapping resistance loci, and validating marker–trait associations in independent populations. In addition, combining host genotyping with pathogen population analyses would further enhance understanding of resistance durability and host–pathogen interactions.

## 5. Conclusions

The genetic basis of resistance to BS and NB was investigated in a diverse panel of 86 two-rowed spring barley accessions from Kazakhstan using SSR-based genotyping and phenotypic evaluation under natural infection across two contrasting environments. Disease expression was strongly environment-dependent. The mean BS severity was 16.3 ± 10.9%, with slightly higher values at KRIAPG (17.8%) than at RIBSP (14.8%). In contrast, NB severity was substantially higher at RIBSP (29.0%) than at KRIAPG (12.2%), with an overall mean of 20.6 ± 9.9%. No significant correlations between locations were detected for either disease, highlighting environmental modulation of resistance. Thirteen polymorphic SSR markers revealed moderate to high genetic diversity (mean PIC = 0.637 ± 0.021; mean alleles per locus = 6.69 ± 0.91). AMOVA indicated that 90% of molecular variance occurred among accessions, and population structure analysis supported clear genetic stratification. MLM analysis identified seven significant (*p* < 0.05) allele–trait associations. The strongest effect was observed for the allele *Bmac209* 176 bp with NB at KRIAPG. The *Bmag206* 246 bp allele showed a protective effect against NB, whereas *Bmag206* 252 bp, *Bmag613* 176 bp, and *HvLEU* 186 bp were associated with increased susceptibility. Regression analysis confirmed additive effects of favorable alleles, particularly for NB. These results provide SSR-based markers for resistance improvement in barley breeding programs in Kazakhstan.

## Figures and Tables

**Table 1:** List of SSR-markers used for genotyping.

SSR	Chromosome	Position (cM) *	Disease	Gene	Ta, °C	Reference
*HVM60*	3H	73.19	BS	*Rrs4*	55	[34]
*Bmac209*	3H	52.39	BS	QTL	58	[35]
*GBM1094*	3H	52.67	BS	QTL	55	[36]
*Bmag112*	3H	65.5	BS	QTL	58	[36]
*Bmag021*	7H	6.78	BS	QTL	58	[37]
*Bmag206*	7H	15.25	BS	QTL	58	[37]
*HVM04*	7H	22.19	BS	QTL	55	[38]
*HVM36*	2H	30.97	NB	*Rpt3*	55	[39]
*Bmag381*	2H	76.22	NB	QTL	60	[40]
*Bmag606*	3H	101.77	NB	*Rpt1*	55	[39]
*HVM03*	4H	58.33	NB	*Rpt8.j*	55	[18]
*HVLEU*	5H	51.26	NB	*Rpt6.g*	55	[41]
*Bmag222*	5H	144.14	NB	QTL	58	[40]
*Bmag613*	6H	69.82	NB	QTL	55	[42]

*—according to Hordeum-Consensus2007-SSR map [43]. Ta—annealing temperature, BS—barley scald, NB—net blotch.

**Table 2:** Phenotypic evaluation of barley resistance to two fungal diseases in two environments.

Stat/Environment/Disease	Barley Scald (%)			Net Blotch (%)		
	KRIAPG 2022	RIBSP 2024	Mean	KRIAPG 2022	RIBSP 2024	Mean
Min	0.0	0.0	0.0	0.0	0.0	0.8
Max	40	50	45.0	30	60	30.3
Mean	17.8	14.8	16.3	12.2	29.0	20.6
SD	9.8	12.0	10.9	5.1	14.6	9.9
CV (%)	55.1	81.1	68.1	41.8	50.3	46.1

KRIAPG—Kazakh Research Institute of Agriculture and Plant Growing (southeastern Kazakhstan), RIBSP—Research Institute of Biological Safety Problems (southern Kazakhstan), CV—coefficient of variation.

**Table 3:** Genetic diversity parameters of SSR markers in the studied barley population.

SSR	Allele Range (bp)	Na	Ne	I	PIC
*HVM03*	154–202	8	5.479	1.837	0.819
*HVM36*	107–119	5	3.221	1.295	0.611
*HVM04*	0–203	4	2.586	1.051	0.688
*HVM60*	114–120	3	1.903	0.822	0.471
*HvLEU*	178–190	4	1.927	0.873	0.505
*Bmag606*	0–142	11	3.897	1.654	0.810
*Bmag021*	0–140	6	3.311	1.337	0.695
*Bmag206*	236–268	12	6.925	2.118	0.854
*Bmac209*	170–194	9	6.278	1.973	0.839
*Bmag613*	148–194	11	4.828	1.934	0.802
*GBM1094*	138–140	2	1.916	0.671	0.476
*Bmag222*	135–177	5	1.274	0.508	0.215
*Bmag112*	158–180	7	1.902	1.035	0.493
Mean ± SE	-	6.692 ± 0.909	3.496 ± 0.514	1.316 ± 0.150	0.637 ± 0.021

Na—number of alleles, Ne—number of effective alleles, I—Shannon’s information index, PIC—polymorphism information content, SE—standard error.

**Table 4:** Genetic diversity indices within barley accessions.

ID	%P	Ne	I	ID	%P	Ne	I
QB_143	7.69	1.06 ± 0.06	0.05 ± 0.05	QB_186	0.00	1.00 ± 0.00	0.00 ± 0.00
QB_144	15.38	1.12 ± 0.08	0.10 ± 0.07	QB_187	0.00	1.00 ± 0.00	0.00 ± 0.00
QB_145	46.15	1.37 ± 0.12	0.29 ± 0.09	QB_188	15.38	1.12 ± 0.08	0.10 ± 0.07
QB_146	7.69	1.06 ± 0.06	0.05 ± 0.05	QB_189	38.46	1.31 ± 0.11	0.24 ± 0.09
QB_147	30.77	1.25 ± 0.11	0.20 ± 0.09	QB_190	15.38	1.12 ± 0.08	0.10 ± 0.07
QB_148	0.00	1.00 ± 0.00	0.00 ± 0.00	QB_191	0.00	1.00 ± 0.00	0.00 ± 0.00
QB_149	15.38	1.12 ± 0.08	0.10 ± 0.07	QB_192	0.00	1.00 ± 0.00	0.00 ± 0.00
QB_150	15.38	1.12 ± 0.08	0.10 ± 0.07	QB_193	15.38	1.12 ± 0.08	0.10 ± 0.07
QB_151	0.00	1.00 ± 0.00	0.00 ± 0.00	QB_194	7.69	1.06 ± 0.06	0.05 ± 0.05
QB_152	0.00	1.00 ± 0.00	0.00 ± 0.00	QB_195	7.69	1.15 ± 0.15	0.08 ± 0.08
QB_153	15.38	1.12 ± 0.08	0.10 ± 0.07	QB_196	7.69	1.06 ± 0.06	0.05 ± 0.05
QB_154	7.69	1.06 ± 0.06	0.05 ± 0.05	QB_197	15.38	1.12 ± 0.08	0.10 ± 0.07
QB_155	7.69	1.06 ± 0.06	0.05 ± 0.05	QB_198	7.69	1.06 ± 0.06	0.05 ± 0.05
QB_156	7.69	1.06 ± 0.06	0.05 ± 0.05	QB_199	15.38	1.12 ± 0.08	0.10 ± 0.07
QB_157	15.38	1.12 ± 0.08	0.10 ± 0.07	QB_200	0.00	1.00 ± 0.00	0.00 ± 0.00
QB_158	15.38	1.12 ± 0.08	0.10 ± 0.07	QB_201	0.00	1.00 ± 0.00	0.00 ± 0.00
QB_159	0.00	1.00 ± 0.00	0.00 ± 0.00	QB_202	15.38	1.22 ± 0.16	0.13 ± 0.09
QB_160	7.69	1.06 ± 0.06	0.05 ± 0.05	QB_203	15.38	1.12 ± 0.08	0.10 ± 0.07
QB_161	7.69	1.06 ± 0.06	0.05 ± 0.05	QB_204	0.00	1.00 ± 0.00	0.00 ± 0.00
QB_162	15.38	1.12 ± 0.08	0.10 ± 0.07	QB_205	0.00	1.00 ± 0.00	0.00 ± 0.00
QB_163	7.69	1.06 ± 0.06	0.05 ± 0.05	QB_206	7.69	1.06 ± 0.06	0.05 ± 0.05
QB_164	23.08	1.18 ± 0.10	0.15 ± 0.08	QB_207	15.38	1.12 ± 0.08	0.10 ± 0.07
QB_165	30.77	1.25 ± 0.11	0.20 ± 0.09	QB_208	7.69	1.06 ± 0.06	0.05 ± 0.05
QB_166	0.00	1.00 ± 0.00	0.00 ± 0.00	QB_209	23.08	1.18 ± 0.10	0.15 ± 0.08
QB_167	7.69	1.06 ± 0.06	0.05 ± 0.05	QB_210	7.69	1.06 ± 0.06	0.05 ± 0.05
QB_168	0.00	1.00 ± 0.00	0.00 ± 0.00	QB_211	15.38	1.12 ± 0.08	0.10 ± 0.07
QB_169	7.69	1.06 ± 0.06	0.05 ± 0.05	QB_212	7.69	1.06 ± 0.06	0.05 ± 0.05
QB_170	15.38	1.12 ± 0.08	0.10 ± 0.07	QB_213	7.69	1.06 ± 0.06	0.05 ± 0.05
QB_171	7.69	1.06 ± 0.06	0.05 ± 0.05	QB_214	15.38	1.12 ± 0.08	0.10 ± 0.07
QB_172	7.69	1.06 ± 0.06	0.05 ± 0.05	QB_215	7.69	1.06 ± 0.06	0.05 ± 0.05
QB_173	0.00	1.06 ± 0.06	0.00 ± 0.00	QB_216	7.69	1.06 ± 0.06	0.05 ± 0.05
QB_174	7.69	1.06 ± 0.06	0.05 ± 0.05	QB_218	0.00	1.00 ± 0.00	0.00 ± 0.00
QB_175	0.00	1.00 ± 0.00	0.00 ± 0.00	QB_219	7.69	1.06 ± 0.06	0.05 ± 0.05
QB_176	0.00	1.00 ± 0.00	0.00 ± 0.00	QB_220	15.38	1.12 ± 0.08	0.10 ± 0.07
QB_177	23.08	1.18 ± 0.10	0.15 ± 0.08	QB_221	0.00	1.00 ± 0.00	0.00 ± 0.00
QB_178	15.38	1.22 ± 0.16	0.13 ± 0.09	QB_222	7.69	1.06 ± 0.06	0.05 ± 0.05
QB_179	15.38	1.22 ± 0.16	0.13 ± 0.09	QB_223	0.00	1.00 ± 0.00	0.00 ± 0.00
QB_180	7.69	1.06 ± 0.06	0.05 ± 0.05	QB_224	7.69	1.06 ± 0.06	0.05 ± 0.05
QB_181	15.38	1.12 ± 0.08	0.10 ± 0.07	QB_225	0.00	1.00 ± 0.00	0.00 ± 0.00
QB_182	23.08	1.18 ± 0.10	0.15 ± 0.08	QB_226	15.38	1.12 ± 0.08	0.10 ± 0.07
QB_183	7.69	1.15 ± 0.15	0.08 ± 0.08	QB_227	7.69	1.06 ± 0.06	0.05 ± 0.05
QB_184	15.38	1.12 ± 0.08	0.10 ± 0.07	QB_228	0.00	1.00 ± 0.00	0.00 ± 0.00
QB_185	7.69	1.06 ± 0.06	0.05 ± 0.05	QB_229	0.00	1.00 ± 0.00	0.00 ± 0.00

%P—% of polymorphic loci, Ne—number of effective alleles, I—Shannon’s information index. Ne and I are presented as mean values ± standard error.

**Table 5:** Significant associations (*p* < 0.05) between SSR markers and disease resistance traits revealed by MLM analysis.

Allele	*p*-Value	Effect on Disease Severity	Disease/Environment
*Bmac209_176* bp	4.477 × 10^−5^	8.307	NB_KRIAPG
*Bmag206_246* bp	0.009	−4.306	NB_mean
*Bmag206_246* bp	0.035	−5.508	NB_RIBSP
*Bmag206_252* bp	0.018	8.284	BS_RIBSP
*Bmag613_176* bp	0.026	2.843	NB_mean
*HvLEU_186* bp	0.009	3.940	BS_mean
*HvLEU_186* bp	0.035	3.941	BS_KRIAPG

BS—barley scald, NB—net blotch, KRIAPG—Kazakh Research Institute of Agriculture and Plant Growing, RIBSP—Research Institute of Biological Safety Problems.

## Data Availability

The original contributions presented in this study are included in the article/Supplementary Materials.

## Supplementary Materials

The following supporting information can be downloaded at https://www.mdpi.com/article/10.3390/genes17030261/s1, Table S1: The list of barley accessions used in the study; Table S2: Meteorological data of vegetation period; Table S3: Genotyping results of 13 SSRs; Figure S1: Mean severity of BS and NB by population structure clusters.

## Abbreviations

The following abbreviations are used in this manuscript:

AMOVA	Analysis of molecular variance
BS	Barley scald
KRIAPG	Kazakh Research Institute of Agriculture and Plant Growing
MLM	Mixed linear model
NB	Net blotch
PIC	Polymorphism information content
RIBSP	Research Institute of Biological Safety Problems
SSR	Simple sequence repeat
