# Supplementary figures and images for: SSR-Based Analysis of Genetic Diversity and Resistance to Barley Scald and Net Blotch in a Collection of Barley from Kazakhstan
Source: Genes (Basel). 2026 Feb 25;17(3):261. doi: 10.3390/genes17030261 (PMC13025345; doi:10.3390/genes17030261)

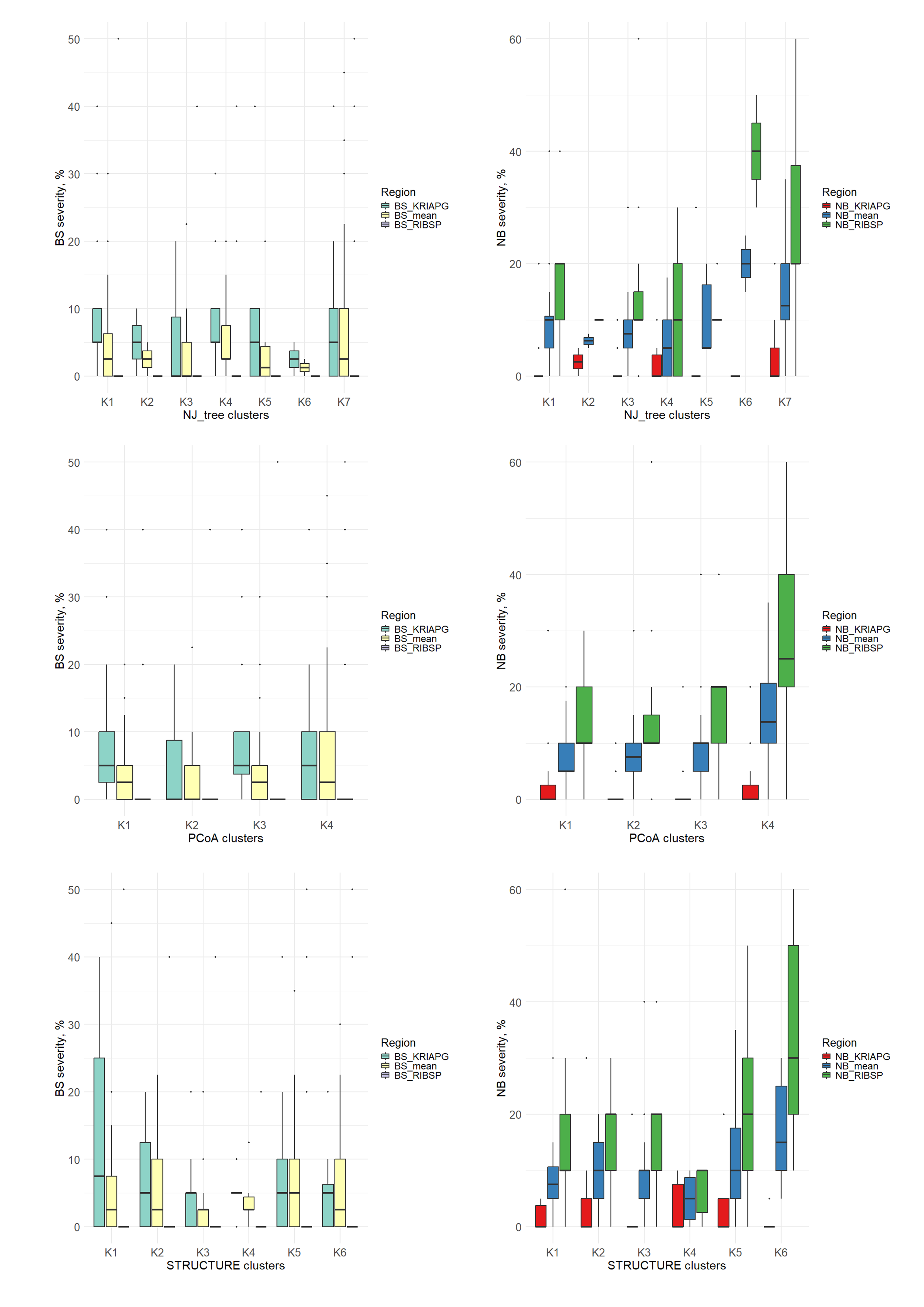

Supplement: Supplementary file 1 [file genes-17-00261-s001.zip › Supplementary Figure S1.png]
